# Physico-technical medicine: teaching technical skills to the intensivist

**DOI:** 10.1186/cc12440

**Published:** 2013-03-19

**Authors:** J Seifried, S Richter, J Guttmann, S Schumann

**Affiliations:** 1 University Medical Center Freiburg, Germany

## Introduction

The requirements for the intensivist in handling medical technology are constantly growing. It appears necessary to acquire technological competences particularly within the fields of medical technology and physics. In the master's degree program 'MasterOnline Physico-Technical Medicine', such technical authority is conveyed. To cope with the intensive vocational situation of the physician, this study course follows the blended-learning concept; that is, it is conceived as an online study course with small portions of intermittent presence phases. Within the first year, technical basic skills such as 'measurement technique', 'informatics', and 'advanced physics' are covered. Subsequently, two of various advanced courses in different fields of medical technology ('technology in intensive care medicine', 'technology in surgery', 'technical cardiology', 'radiology', and other) are selected.

## Methods

In a survey, we evaluated the study course. Therefore, a questionnaire was distributed among all students including the topics course contents, learning materials, time management, supervision, and overall impression. The students were asked to score their agreement to the statements 'content is well structured', 'content extent is appropriate' and 'content is relevant for medical purposes' on a scale ranging from 1 (fully disagree) to 5 (fully agree).

## Results

The students participated actively in this study course with highest motivation and large commitment. The students' workload was in the targeted range of about 10 hours/week. Content structure was scored with 4.3 ± 0.1, content extent with 4.1 ± 0.2 and medical relevance with 4.3 ± 0.2.

## Conclusion

The blended-learning concept fulfills the requirements for occupation-accompanying continued medical education, since it offers the possibility to study self-employed accessing text documents, lecture recordings, and electronic lectures and to convert in concentrated presence phases this knowledge into practical exercises.

